# County-Level Income Inequality, Social Mobility, and Deaths of Despair in the US, 2000-2019

**DOI:** 10.1001/jamanetworkopen.2023.23030

**Published:** 2023-07-12

**Authors:** Chun-Tung Kuo, Ichiro Kawachi

**Affiliations:** 1 Department of Social and Behavioral Sciences, Harvard T.H. Chan School of Public Health, Boston, Massachusetts; 2 Institute of Health Behaviors and Community Sciences, College of Public Health, National Taiwan University, Taipei, Taiwan

## Abstract

**Importance:**

Increasing mortality from suicide, drug overdose, and alcohol-related liver disease (collectively referred to as *deaths of despair*) is a critical public health crisis. Income inequality and social mobility have been separately associated with all-cause mortality; however, no studies have examined their interaction with these preventable deaths.

**Objective:**

To assess the interaction between income inequality and social mobility with deaths of despair among working-age Hispanic, non-Hispanic Black, and non-Hispanic White populations.

**Design, Setting, and Participants:**

This cross-sectional study analyzed data from the Centers for Disease Control and Prevention WONDER (Wide-Ranging Online Data for Epidemiologic Research) database on county-level deaths of despair from 2000 to 2019 across racial and ethnic groups. Statistical analysis was performed from January 8 to May 20, 2023.

**Exposures:**

The primary exposure of interest was county-level income inequality, measured by the Gini coefficient. Another exposure was race- and ethnicity-specific absolute social mobility. Tertiles for the Gini coefficient and social mobility were created to evaluate the dose-response association.

**Main Outcomes and Measures:**

The main outcomes were adjusted risk ratios (RRs) of deaths from suicide, drug overdose, and alcoholic liver disease. The interaction between income inequality and social mobility was formally tested on both the additive and multiplicative scales.

**Results:**

The sample included 788 counties for Hispanic populations, 1050 counties for non-Hispanic Black populations, and 2942 counties for non-Hispanic White populations. Over the study period, 152 350, 149 589, and 1 250 156 deaths of despair were recorded for working-age Hispanic, non-Hispanic Black, and non-Hispanic White populations, respectively. Compared with the reference group (counties with low income inequality and high social mobility), counties with greater income inequality (high inequality: RR, 1.26 [95% CI, 1.24-1.29] for Hispanic populations; RR, 1.18 [95% CI, 1.15-1.20] for non-Hispanic Black populations; and RR, 1.22 [95% CI, 1.21-1.23] for non-Hispanic White populations) or less social mobility (low mobility: RR, 1.79 [95% CI, 1.76-1.82] for Hispanic populations; RR, 1.64 [95% CI, 1.61-1.67] for non-Hispanic Black populations; and RR, 1.38 [95% CI, 1.38-1.39] for non-Hispanic White populations) had higher RRs for deaths of despair. In counties with high income inequality and low social mobility, positive interactions were observed on the additive scale for Hispanic populations (relative excess risk due to interaction [RERI], 0.27 [95% CI, 0.17-0.37]), non-Hispanic Black populations (RERI, 0.36 [95% CI, 0.30-0.42]), and non-Hispanic White populations (RERI, 0.10 [95% CI, 0.09-0.12]). In contrast, positive interactions on the multiplicative scale were found only for non-Hispanic Black populations (ratio of RRs, 1.24 [95% CI, 1.18-1.31]) and non-Hispanic White populations (ratio of RRs, 1.03 [95% CI, 1.02-1.05]), but not for Hispanic populations (ratio of RRs, 0.98 [95% CI, 0.93-1.04]). In the sensitivity analyses using continuous Gini coefficient and social mobility, a positive interaction was observed between higher income inequality and lower social mobility with deaths of despair on both the additive and multiplicative scales for all 3 racial and ethnic groups.

**Conclusions and Relevance:**

This cross-sectional study found that the joint exposure of unequal income distribution and lack of social mobility was associated with additional risks for deaths of despair, suggesting that addressing the underlying social and economic conditions is crucial in responding to the epidemic of deaths of despair.

## Key Points

**Question:**

Is the interaction between income inequality and social mobility associated with an increased risk of deaths of despair (deaths from suicide, drug overdose, and alcoholic liver disease) among the working-age population in the US?

**Findings:**

In this cross-sectional study, higher income inequality and lower social mobility were associated with a higher burden of deaths of despair for Black, Hispanic, and White populations. In addition, the joint exposure of unequal income distribution and lack of social mobility was associated with additional risks of deaths of despair on both the additive and multiplicative scales.

**Meaning:**

The findings of this study suggest that policy responses to the epidemic of deaths of despair must address the underlying social and economic conditions associated with these deaths.

## Teaser

This cross-sectional study uses US county-level data to assess the association of the joint exposure of income inequality and social mobility with death from suicide, drug overdose, and alcohol-related liver disease among working-age Hispanic, non-Hispanic Black, and non-Hispanic White populations.

## Introduction

Life expectancy at birth in the US has sharply decreased from 78.8 years in 2019 to 76.1 years in 2021, driven by the COVID-19 pandemic, which brought the country’s life expectancy back to the levels of 1996.^1^ Differences in the decrease in life expectancy between racial and ethnic groups have also been observed.^2,3^ Even before the pandemic, life expectancy in the US had been decreasing for 3 consecutive years since 2015, contrary to the trend observed in all other developed countries in the Organisation for Economic Co-operation and Development.^4^ This phenomenon has been associated largely with the increase in mortality among middle-aged non-Hispanic White individuals (hereafter, *White*) in the US since 1999.^5^ Case and Deaton^6,7^ coined the term *deaths of despair* to describe the contributing causes of mortality, namely, deaths from suicide, alcohol abuse, and drug overdose (the opioid crisis in the US). These cause-specific deaths are also trending high among non-Hispanic Black (hereafter, *Black*) and Hispanic populations in recent years^8,9^ and even continued to increase during the COVID-19 pandemic.^10,11^

The increase in deaths of despair is attributed to a constellation of self-destructive health behaviors (alcohol and drug abuse and suicide) that are associated with communities’ underlying social and economic conditions.^12,13^ A national report also found that the epidemic of opioid overdose deaths, which began in rural areas in 2000, has become a national phenomenon in the US.^14^ Although national discourse on responding to the opioid crisis has focused on issues such as the urgent need to expand treatment and rehabilitation services, many have pointed out the need to look upstream (ie, understanding the social and economic circumstances that make people despair).^15,16,17^

Income inequality has been put forward as a crucial social problem of our time.^18^ Ecological and multilevel research in the US has revealed that states with greater income inequality have worse population health.^19^ One crucial mechanism linking income inequality to health is psychosocial stress. When the gap between those with ample income and those with insufficient income widens in society, those left behind, even those who may not be deprived in an absolute sense (ie, lacking sufficient food, nutrition, living space, or safe neighborhoods) experience a sense of relative deprivation. This psychosocial deprivation can directly harm mental well-being (feelings of self-worth and hope) and/or increase the risk of harmful coping behaviors in an attempt to alleviate psychological stress.^19^ Studies of relative deprivation have linked it to depressive symptoms, cigarette smoking, and alcohol consumption.^20,21,22^ Individuals can also be relatively deprived in a material sense (eg, lacking access to high-speed internet and therefore the ability to telecommute during the pandemic or not being able to afford to turn on the air conditioner during heat waves). Another mechanism posits that income inequality erodes social capital and social cohesion.^23,24^ Lower levels of social capital have been associated with higher rates of suicide and drug overdose mortality.^25,26^ Therefore, during a period of increasing income inequality, it is plausible to hypothesize an increase in population harms associated with self-destructive health behaviors, such as drug abuse, alcohol poisonings, and suicide,^27^ although only limited research has tested this association, to our knowledge.^28^

Separate from the literature on the population health consequences of income inequality, a growing body of studies has implicated lack of social and economic mobility as a critical determinant of health in the US.^29,30,31^ Income inequality and social mobility are inversely related. Thus, higher levels of income inequality have been associated with less social mobility.^32^ Parallel to the increase in income inequality since the 1980s,^33^ absolute income mobility (children who earn more than their parents) has decreased among recent cohorts, and the “American Dream” (the belief that hard work and opportunity will lead to a better life) is fading away.^34^ Using intergenerational mobility as a measure of social mobility,^35^ previous studies have found that economic opportunity is inversely associated with all-cause mortality, self-reported health, and health behaviors in the US.^29,30^ A study found that low economic opportunity at the US county level was associated with increased all-cause mortality for White adults aged 45 to 54 years between 2010 and 2012.^31^ In addition, a cross-sectional study found that higher county-level social mobility was associated with smaller income-related gaps in life expectancy in the US.^36^

Although income inequality and social mobility have been separately associated with population health, no studies have investigated the joint association of income inequality and social mobility with deaths of despair, to our knowledge. Recent literature has proposed focusing on the interactions between income inequality, social mobility, and population health as an important research agenda.^37^ The literature has also suggested that linking income inequality to cause-specific mortality instead of all-cause mortality, particularly avoidable and preventable mortality, could provide significant implications for policy responses.^37^ We thus addressed this knowledge gap by examining the interaction between county-level income inequality and social mobility with deaths of despair (suicide, drug overdose, and alcoholic liver disease) among working-age Black, Hispanic, and White populations.

## Methods

### Data Sources

Data on county-level, cause-specific and race- and ethnicity-specific numbers of deaths and population size from 2000 to 2019 were extracted from the Centers for Disease Control and Prevention WONDER (Wide-Ranging Online Data for Epidemiologic Research) database.^38^ In the database, statistics representing fewer than 10 individuals are suppressed. We used aggregated data over a 20-year period to ensure that a larger number of counties could provide reliable cause-specific numbers of deaths from the database for 3 racial and ethnic groups: Black, Hispanic, and White populations. The county-level Gini coefficient and sociodemographic characteristics for 2000 were obtained from the US Census Bureau.^39^ Also, we obtained the race- and ethnicity-specific social mobility indicators from a publicly available online source, the Opportunity Atlas data library.^40^ This study used solely publicly available aggregate data; therefore, no ethical approval was sought for the study procedures. We followed the Strengthening the Reporting of Observational Studies in Epidemiology (STROBE) reporting guideline.

### Measurement of Deaths of Despair

The outcome variable was the county-level and race- and ethnicity-specific mortality caused by deaths of despair among working-age individuals in the US from 2000 to 2019. We followed previous studies^5,6^ and used *International Statistical Classification of Diseases and Related Health Problems, Tenth Revision* codes to categorize causes of death into 3 categories: suicide (codes X60-84 and Y87.0), drugs and alcohol poisoning (codes X40-45, Y10-15, Y45, Y47, and Y49), and alcohol-related liver diseases and cirrhosis (codes K70 and K73-74).

### Measurement of Income Inequality

The primary exposure of interest was county-level income inequality, measured by the Gini coefficient of household disposable income for the year 2000, which was obtained from the US Census Bureau.^41^ The Gini coefficient is a widely used summary measure for the degree of income inequality, with a value ranging between 0 (perfectly equal) and 1 (perfectly unequal).

### Measurement of Social Mobility

We used the calculations of race- and ethnicity-specific absolute upward mobility of Chetty and colleagues^40^ as the measure of intergenerational social mobility for each county. Absolute social mobility was defined as the mean household income rank (measured in 2014-2015) of children born between 1978 and 1983 in the US whose parents were at the 25th percentile of the national income distribution (measured in 1994-2000), based on an ordinary least-square regression line of child rank on parent rank. Chetty and colleagues^40^ analyzed data on 20.5 million children, which covered 96% of the target population, in 1978 to 1983 birth cohorts. Children’s incomes were calculated using their mean earnings in 2014 to 2015, when they were between the ages of 31 and 37 years. Absolute social mobility ranges from 0 to 1, with a higher value indicating greater intergenerational economic mobility.

### Other Covariates

Following prior research on the association between social mobility and mortality,^29,31^ this study controlled for several sociodemographic characteristics. To account for potential confounding of linking income inequality and social mobility to race- and ethnicity-specific mortality risks, we adjusted for county-level mean income, the proportion of higher education attainment, and unemployment rates in 2000 for each racial and ethnic group. We also controlled for total population density in 2000 as an indicator of county socioeconomic development.

### Statistical Analysis

Statistical analysis was performed from January 8 to May 20, 2023. We used Poisson regression to analyze the association of income inequality and social mobility with deaths of despair. The logarithm of the number of deaths was the outcome variable, and the logarithm of the population size was included as an offset term. We estimated the mortality risk ratios (RRs) using the following model: log (*y_i_*) = α + β_1_ × *x*_1_*_i_* + β_2_ × *x*_2_*_i_* + γ × *c_i_* + log (population*_i_*) + ε*_i_*, where *y_i_* is the number of deaths of despair for county *i*; *x*_1_*_i_* and *x*_2_*_i_* are vectors of income inequality and social mobility for county *i*; and *c_i_* is a vector of covariates. Population*_i_* is the working-age population size of each county.

To evaluate the dose-response association, we created tertiles for the Gini coefficient and social mobility. The reference group was county with low income inequality and high social mobility. First, we estimated adjusted RRs for the main association of income inequality and social mobility with mortality while controlling for all covariates. Then, to assess the interaction, we estimated RRs for the joint exposure of income inequality and social mobility and calculated measures of interaction on both the additive and multiplicative scales.^42^ The additive interactions were estimated by relative excess risk due to interaction (RERI): RERI_11_ = RR_11_ − RR_10_ − RR_01_ + 1, where subscript 1 represents a certain stratum for income inequality (medium or high inequality) or social mobility (medium or low mobility), while subscript 0 represents the reference group of income inequality (low inequality) or social mobility (high mobility). A positive RERI value indicates a positive interaction, a negative value indicates a negative interaction, and a RERI value of 0 indicates no additive interaction. On the other hand, multiplicative interactions were estimated by the ratio of RRs (RRR) using the following formula: RRR_11_ = RR_11_/(RR_10_ × RR_01_). An RRR value of 1 indicates no multiplicative interaction, an RRR value greater than 1 indicates a positive interaction, and an RRR value less than 1 indicates a negative interaction.^42,43^

To check robustness, we conducted a sensitivity analysis by coding the Gini coefficient and absolute social mobility as continuous variables in the Poisson regression model. To facilitate interpretation, absolute social mobility was inversely calculated as social immobility (1 − absolute social mobility). We then created a product term between income inequality and social immobility (both standardized to avoid multicollinearity) and conducted tests for interaction on the additive and multiplicative scales. In addition, we analyzed the association of income inequality and social mobility with each cause of death for White populations as a sensitivity analysis. We were unable to perform this analysis for Black and Hispanic populations because most county-level mortality data were suppressed. All statistical analyses were conducted with Stata, version 17.0 (StataCorp LP). All *P* values were from 2-sided tests and results were deemed statistically significant at *P* < .05.

## Results

Of 3142 US counties, our sample included 788 counties for Hispanic populations, 1050 counties for Black populations, and 2942 counties for White populations, representing 95.5%, 96.5%, and 99.8% of the population for each racial and ethnic group, respectively. Over the study period, 149 589, 152 350, and 1 250 156 deaths of despair were recorded for working-age Black, Hispanic, and White populations, respectively. County-level income inequality, social mobility, and sociodemographic characteristics for 3 racial and ethnic groups are shown in Table 1. The mean Gini coefficients were similar across racial and ethnic groups, whereas the absolute social mobility for Black populations was substantially lower than for Hispanic and White populations. The Gini coefficient was negatively correlated with absolute social mobility for Black (*r* = −0.24; *P* < .001) and White (*r* = −0.19; *P* < .001) populations, but not for Hispanic populations (*r* = −0.02; *P* = .66).

**Table 1.** County-Level Sociodemographic Characteristics

Variable	Mean (SD) value		
	Hispanic (n = 788)	Non-Hispanic Black (n = 1050)	Non-Hispanic White (n = 2942)
Income inequality			
Gini coefficient	0.44 (0.04)	0.45 (0.04)	0.43 (0.04)
Social mobility			
Absolute social mobility^a^	0.42 (0.04)	0.33 (0.03)	0.46 (0.05)
Sociodemographic variables			
Income per capita (USD), $^a^	11 970 (3067)	13 129 (4037)	19 342 (4741)
Some college or above, %^a^	31.7 (13.4)	36.6 (14.5)	45.3 (12.2)
Unemployment, %^a^	8.9 (3.8)	11.6 (4.4)	4.8 (2.1)
Population density, log	4.9 (1.9)	5.1 (1.4)	3.9 (1.6)

^a^ Race- and ethnicity-specific characteristic.

For the main association of the exposure of interest (Table 2), we observed higher mortality RRs for deaths of despair in counties with greater income inequality (high inequality: RR, 1.18 [95% CI, 1.15-1.20] for Black populations; RR, 1.26 [95% CI, 1.24-1.29] for Hispanic populations; and RR, 1.22 [95% CI, 1.21-1.23] for White populations) or less social mobility (low mobility: RR, 1.64 [95% CI, 1.61-1.67] for Black populations; RR, 1.79 [95% CI, 1.76-1.82] for Hispanic populations; and RR, 1.38 [95% CI, 1.38-1.39] for White populations). The monotonic gradient patterns were also observed for each cause of death from suicide, drug overdose, and alcoholic liver disease among White populations (eTable 1 in Supplement 1).

**Table 2.** Adjusted Estimates of the Main Association of Income Inequality and Social Mobility With Deaths of Despair

Characteristic	ARR (95% CI)		
	Hispanic counties (n = 788)	Non-Hispanic Black counties (n = 1050)	Non-Hispanic White counties (n = 2942)
Income inequality			
Low	1 [Reference]	1 [Reference]	1 [Reference]
Medium	1.03 (1.01-1.05)	1.14 (1.12-1.16)	1.10 (1.10-1.11)
High	1.26 (1.24-1.29)	1.18 (1.15-1.20)	1.22 (1.21-1.23)
Social mobility			
High	1 [Reference]	1 [Reference]	1 [Reference]
Medium	1.25 (1.24-1.27)	1.44 (1.42-1.46)	1.20 (1.19-1.21)
Low	1.79 (1.76-1.82)	1.64 (1.61-1.67)	1.38 (1.38-1.39)

Abbreviation: ARR, adjusted risk ratio (adjusting for all covariates).

Table 3 shows associations of the joint exposure of income inequality and social mobility with deaths of despair, as well as corresponding measures of interaction. Compared with the reference group (counties with low income inequality and high social mobility), counties with greater income inequality or less social mobility had higher adjusted RRs for deaths of despair. The highest RRs were observed in counties with high income inequality and low social mobility for Hispanic populations (RR, 2.56 [95% CI, 2.47-2.66]) and White populations (RR, 1.60 [95% CI, 1.58-1.61]) and with medium income inequality and low social mobility for Black populations (1.80 [95% CI, 1.75-1.86]). For Black and White populations, counties with high income inequality and medium or low social mobility had positive interactions on both the additive and multiplicative scales. For counties with high income inequality and low social mobility, the RERI was 0.36 (95% CI, 0.30-0.42) and the RRR was 1.24 (95% CI, 1.18-1.31) for Black populations, and the RERI was 0.10 (95% CI, 0.09-0.12) and the RRR was 1.03 (95% CI, 1.02-1.05) for White populations. For Hispanic populations, counties with high income inequality and low social mobility had a positive interaction on the additive scale (RERI, 0.27 [95% CI, 0.17-0.37]) but not on the multiplicative scale (RRR, 0.98 [95% CI, 0.93-1.04]).

**Table 3.** Additive and Multiplicative Interactions of Income Inequality and Social Mobility With Deaths of Despair

Social mobility	Income inequality, ARR (95% CI)		
	Low inequality	Medium inequality	High inequality
**Hispanic counties (n = 788)**			
High mobility	1 [Reference]	1.25 (1.20 to 1.29)	1.32 (1.28 to 1.37)
Medium mobility	1.42 (1.36 to 1.47)	1.34 (1.30 to 1.39)	1.71 (1.66 to 1.77)
Low mobility	1.97 (1.87 to 2.08)	1.79 (1.72 to 1.86)	2.56 (2.47 to 2.66)
Interaction			
RERI (additive scale)			
Medium mobility	Reference	−0.32 (−0.38 to −0.26)	−0.03 (−0.08 to 0.02)
Low mobility	Reference	−0.42 (−0.53 to −0.31)	0.27 (0.17 to 0.37)
RRR (multiplicative scale)			
Medium mobility	Reference	0.76 (0.73 to 0.79)	0.91 (0.88 to 0.95)
Low mobility	Reference	0.73 (0.69 to 0.78)	0.98 (0.93 to 1.04)
**Non-Hispanic Black counties (n = 1050)**			
High mobility	1 [Reference]	0.99 (0.96 to 1.03)	1.05 (1.02 to 1.09)
Medium mobility	1.25 (1.21 to 1.30)	1.46 (1.42 to 1.51)	1.58 (1.54 to 1.63)
Low mobility	1.34 (1.28 to 1.41)	1.80 (1.75 to 1.86)	1.75 (1.70 to 1.81)
Interaction			
RERI (additive scale)			
Medium mobility	Reference	0.21 (0.16 to 0.26)	0.28 (0.23 to 0.32)
Low mobility	Reference	0.47 (0.40 to 0.54)	0.36 (0.30 to 0.42)
RRR (multiplicative scale)			
Medium mobility	Reference	1.17 (1.12 to 1.23)	1.20 (1.15 to 1.25)
Low mobility	Reference	1.36 (1.28 to 1.43)	1.24 (1.18 to 1.31)
**Non-Hispanic White counties (n = 2942)**			
High mobility	1 [Reference]	1.14 (1.12 to 1.15)	1.14 (1.13 to 1.15)
Medium mobility	1.16 (1.15 to 1.17)	1.24 (1.22 to 1.25)	1.53 (1.52 to 1.54)
Low mobility	1.36 (1.34 to 1.37)	1.53 (1.51 to 1.54)	1.60 (1.58 to 1.61)
Interaction			
RERI (additive scale)			
Medium mobility	Reference	−0.06 (−0.07 to −0.04)	0.24 (0.22 to 0.25)
Low mobility	Reference	0.03 (0.02 to 0.05)	0.10 (0.09 to 0.12)
RRR (multiplicative scale)			
Medium mobility	Reference	0.94 (0.93 to 0.95)	1.16 (1.15 to 1.18)
Low mobility	Reference	0.99 (0.98 to 1.00)	1.03 (1.02 to 1.05)

Abbreviations: ARR, adjusted risk ratio (adjusting for all covariates); RERI, relative excess risk due to interaction (interaction on additive scale); RRR, ratio of risk ratio (interaction on multiplicative scale).

### Sensitivity Analysis

In the analysis for each cause of death among White populations, counties with high income inequality and low social mobility had the highest RRs for suicide (1.37 [95% CI, 1.35-1.39]), drug overdose (1.67 [95% CI, 1.64-1.69]), and alcoholic liver disease (1.86 [95% CI, 1.83-1.90]) (eTable 2 in Supplement 1). For each cause of death, counties with high income inequality and medium or low social mobility also exhibited positive interactions on both additive and multiplicative scales, except for alcoholic liver disease in counties with high income inequality and low social mobility.

In the sensitivity analyses using continuous variables (Table 4), we observed a positive interaction between income inequality and social immobility with deaths of despair on both the additive and multiplicative scales. The RERI was 0.12 (95% CI, 0.10-0.14) and the RRR was 1.04 (95% CI, 1.03-1.05) for the Hispanic population, the RERI was 0.08 (95% CI, 0.06-0.09) and the RRR was 1.06 (95% CI, 1.05-1.07) for the Black population, and the RERI was 0.06 (95% CI, 0.06-0.06) and the RRR was 1.03 (95% CI, 1.03-1.03) for the White population. For each cause of death among White populations, positive interactions on both additive and multiplicative scales were also observed for 3 causes of death (eTable 3 in Supplement 1). The RERI was 0.02 (95% CI, 0.02-0.03) and the RRR was 1.01 (95% CI, 1.00-1.01) for suicide, the RERI was 0.11 (95% CI, 0.10-0.12) and the RRR was 1.07 (95% CI, 1.06-1.07) for drug overdose, and the RERI was 0.06 (95% CI, 0.06-0.07) and the RRR was 1.02 (95% CI, 1.02-1.03) for alcoholic liver disease.

**Table 4.** Interactions of Continuous Income Inequality and Social Immobility With Deaths of Despair

Characteristic	ARR (95% CI)		
	Hispanic (788 counties)	Non-Hispanic Black (1050 counties)	Non-Hispanic White (2942 counties)
Income inequality	1.11 (1.10-1.12)	0.99 (0.99-1.00)	1.09 (1.09-1.09)
Social immobility^a^	1.46 (1.44-1.48)	1.37 (1.35-1.39)	1.22 (1.22-1.22)
Product term	1.04 (1.03-1.05)	1.06 (1.05-1.07)	1.03 (1.03-1.03)
Interaction			
RERI (additive scale)	0.12 (0.10-0.14)	0.08 (0.06-0.09)	0.06 (0.06-0.06)
RRR (multiplicative scale)	1.04 (1.03-1.05)	1.06 (1.05-1.07)	1.03 (1.03-1.03)

Abbreviations: ARR, adjusted risk ratio (adjusting for all covariates); RERI, relative excess risk due to interaction (interaction on additive scale); RRR, ratio of risk ratio (interaction on multiplicative scale).

^a^ Social mobility was inversely calculated as social immobility (calculated as 100 − absolute social mobility).

## Discussion

This study used US county-level data and showed that both income inequality and social mobility are independently associated with deaths of despair among working-age Black, Hispanic, and White populations. Furthermore, the joint exposure of unequal income distribution and social immobility was associated with additional risks for deaths of despair on both the additive and multiplicative scales. To our knowledge, this is the first study that explicitly tests the interaction of income inequality and social mobility with deaths of despair across racial and ethnic groups in the US.

Our findings are in line with prior studies linking income inequality to population health in the US. Previous research suggests that unequal income distribution is associated with age-adjusted drug overdose mortality from narcotic and hallucinogen poisoning at the state level,^44^ as well as higher rates of drug overdose fatality in New York City neighborhoods.^45^ Previous work has found mixed results for alcohol-related liver diseases or alcohol poisoning. In New York City neighborhoods, unequal income distribution was found to be correlated with more alcohol use.^46^ On the other hand, state-level income inequality was not correlated with alcohol dependence rates.^47^ Another study based on the National Alcohol Survey in 2000 and 2005 found that the state-level Gini coefficient was not associated with light or heavy alcohol use or with alcohol-related consequences.^48^ However, a study found that, compared with more egalitarian states, the socioeconomic gradients linking county-level income to mortality from alcoholic liver disease tend to be steeper in more unequal states.^49^ Similarly, the association of income inequality with suicide is inconsistent. One study did not find an association between state-level income inequality and state-level suicide mortality in the 50 US states,^25^ while another work used fixed-effects analysis and instrumental variable analysis and found that higher state-level income inequality was associated with higher individual risk of dying from suicide in the 48 contiguous US states.^50^

Our findings are also coherent with prior studies linking economic opportunity to all-cause mortality and health risks in the US. Venkataramani and colleagues^29,30^ used absolute upward mobility and found that intergenerational social mobility is independently associated with all-cause mortality and health behaviors. Another study of US county-level, all-cause mortality among White individuals aged 45 to 54 years found that relative social mobility was associated with increased mortality between 2010 and 2012.^31^ However, that study did not examine cause-specific mortality and focused solely on the middle-aged White population. In addition, a cross-sectional study of relative social mobility found that greater economic mobility was associated with narrower gaps in life expectancy by income quartile.^36^ Research indicates that relative social mobility is similar for all racial and ethnic groups in the US, while there are disparities in the level of absolute social mobility.^51^ For example, there is a substantial difference in absolute upward mobility, but not in relative social mobility, between Black and White individuals in the US.^51^ The current study contributes to the literature by demonstrating the synergy between higher income inequality and lower absolute social mobility and its association with deaths of despair for Black, Hispanic, and White populations.

A potential mechanism for the positive association of unequal income distribution and low social mobility with mortality is that, in places where individuals born into families with relatively low income cannot obtain economic opportunities, the negative psychosocial effects (eg, shame, frustration, and anxiety) induced by unequal societies may become more profound compared with more egalitarian societies. Both income inequality and limited social mobility might be operating to produce psychosocial stress and contribute to worse health through status insecurity, worries about self-worth, and feelings of inferiority. Although income inequality and social mobility are interrelated (as illustrated by the Great Gatsby curve^32^), intergenerational mobility is derived from the distribution of income in the general population and has less to do with the concentration of wealth among the top 1%. Stagnant economic mobility may exacerbate the psychosocial harm caused by unequal societies in which relatively lower-income populations have little chance to improve income and social mobility.

### Strengths and Limitations

This study has some strengths, including use of a larger number of US counties in the analysis than previous works of which we are aware, focus on avoidable and preventable causes of death rather than all-cause mortality, use of race- and ethnicity-specific social mobility measures for the race- and ethnicity-specific outcomes, and performance of formal interaction tests on both the additive and multiplicative scales.

This study also has some limitations. First, we were unable to establish causality due to the observational and cross-sectional design. A more robust study design would be to investigate the association between changes in economic characteristics and changes in mortality using panel data. Second, we combined 20-year mortality data, which cannot account for the dynamic nature and temporal variations in deaths of despair; for instance, the drug overdose epidemic shifted from prescription opioid–related deaths in middle-class White communities to heroin- and then fentanyl-related dealths, affecting a wider segment of the community.^52^ Data were pooled across years because the annual, or even 5-year, county-level numbers of deaths of despair were mostly suppressed (<10 deaths), particularly in less-populous counties. Third, we cannot account for the association of social and health policies with deaths of despair, such as Medicaid Expansion, prescription drug monitoring programs, state policies on alcohol sales, state-level gun laws, access to health care, and other random spatial effects at the state level. Fourth, the Gini coefficient and covariates were measured in 2000, and these variables may be time varying. However, research suggests that income inequality may have decades-long associations with mortality.^19,53^ Fifth, we did not use the race- and ethnicity-specific Gini coefficient when analyzing the number of deaths for each racial and ethnic group. However, research indicates that income inequality could produce toxic spillover effects, causing adverse health consequences for all people living in unequal societies.^19^

## Conclusions

This cross-sectional study found that the joint exposure of unequal income distribution and lack of social mobility was associated with additional risks for death from suicide, drug overdose, and alcohol-related liver disease among working-age Black, Hispanic, and White individuals in the US, suggesting that, in addition to focusing on the proximal responses to the opioid crisis (eg, expanding addiction treatment and curbing the supply of drugs), addressing the underlying socioeconomic conditions is crucial in responding to the epidemic of deaths of despair.

## Notes

Supplement 1. **eTable 1.** Adjusted Estimates of the Main Association of Income Inequality and Social Mobility With Cause-Specific Deaths Among the Non-Hispanic White Population **eTable 2.** Additive and Multiplicative Interactions of Income Inequality and Social Mobility With Cause-Specific Deaths Among the Non-Hispanic White Population **eTable 3.** Interactions of Continuous Income Inequality and Social Immobility on Cause-Specific Deaths Among the Non-Hispanic White Population

Supplement 2.
Data Sharing Statement
