# Supplementary material for: County-Level Income Inequality, Social Mobility, and Deaths of Despair in the US, 2000-2019
Source: JAMA Netw Open. 2023 Jul 12;6(7):e2323030. doi: 10.1001/jamanetworkopen.2023.23030 (PMC10339154; doi:10.1001/jamanetworkopen.2023.23030)
Supplement: Supplement 1. — eTable 1. Adjusted Estimates of the Main Association of Income Inequality and Social Mobility With Cause-Specific Deaths Among the Non-Hispanic White Population eTable 2. Additive and Multiplicative Interactions of Income Inequality and Social Mobility With Cause-Specific Deaths Among the Non-Hispanic White Population eTable 3. Interactions of Continuous Income Inequality and Social Immobility on Cause-Specific Deaths Among the Non-Hispanic White Population [file jamanetwopen-e2323030-s001.pdf]

Supplementary Online Content

Kuo CT, Kawachi I. County-level income inequality, social mobility, and deaths of despair in the US, 2000-2019. *JAMA Netw Open.* 2023;6(7):e2323030. doi:10.1001/jamanetworkopen.2023.23030

eTable 1. Adjusted Estimates of the Main Association of Income Inequality and Social Mobility With Cause-Specific Deaths Among the Non-Hispanic White Population
eTable 2. Additive and Multiplicative Interactions of Income Inequality and Social Mobility With Cause-Specific Deaths Among the Non-Hispanic White Population
eTable 3. Interactions of Continuous Income Inequality and Social Immobility With Cause-Specific Deaths Among the Non-Hispanic White Population

This supplementary material has been provided by the authors to give readers additional information about their work.

© 2023 Kuo CT et al. *JAMA Network Open.*

eTable 1. Adjusted Estimates of the Main Association of Income Inequality and Social Mobility With Cause-Specific Deaths Among the Non-Hispanic White Population

	ARR (95% CI)		
**Characteristic**	**Suicide (n = 2750)**	**Drug overdose (n = 2438)**	**Alcoholic liver disease (n = 2473)**
**Income inequality**			
Low	1 [Reference]	1 [Reference]	1 [Reference]
Medium	1.07 (1.06–1.07)	1.08 (1.07–1.09)	1.21 (1.20–1.22)
High	1.15 (1.14–1.16)	1.20 (1.19–1.21)	1.36 (1.35–1.38)
**Social mobility**			
High	1 [Reference]	1 [Reference]	1 [Reference]
Medium	1.13 (1.12–1.14)	1.27 (1.26–1.28)	1.21 (1.19–1.22)
Low	1.27 (1.26–1.28)	1.50 (1.48–1.51)	1.39 (1.38–1.41)

Abbreviation: ARR, adjusted risk ratio (adjusting for all covariates).

© 2023 Kuo CT et al. *JAMA Network Open.*

eTable 2. Additive and Multiplicative Interactions of Income Inequality and Social Mobility With Cause-Specific Deaths Among the Non-Hispanic White Population

	**Income inequality, ARR (95% CI)**		
**Social mobility**	**Low inequality**	**Medium inequality**	**High inequality**
**Suicide (n = 2750)**			
High mobility	1 [Reference]	1.06 (1.05 to 1.08)	1.05 (1.03 to 1.07)
Medium mobility	1.08 (1.07 to 1.10)	1.11 (1.09 to 1.12)	1.34 (1.32 to 1.35)
Low mobility	1.20 (1.18 to 1.22)	1.34 (1.32 to 1.36)	1.37 (1.35 to 1.39)
**Interaction**			
RERI (additive scale)			
Medium mobility	Reference	-0.04 (-0.06 to -0.02)	0.20 (0.18 to 0.22)
Low mobility	Reference	0.08 (0.05 to 0.10)	0.12 (0.09 to 0.14)
RRR (multiplicative scale)			
Medium mobility	Reference	0.96 (0.95 to 0.98)	1.18 (1.15 to 1.20)
Low mobility	Reference	1.05 (1.03 to 1.07)	1.08 (1.06 to 1.11)
**Drug overdose (n = 2438)**			
High mobility	1 [Reference]	1.10 (1.09 to 1.12)	1.07 (1.05 to 1.09)
Medium mobility	1.17 (1.16 to 1.19)	1.25 (1.23 to 1.26)	1.57 (1.55 to 1.59)
Low mobility	1.45 (1.43 to 1.48)	1.56 (1.54 to 1.58)	1.67 (1.64 to 1.69)
**Interaction**			
RERI (additive scale)			
Medium mobility	Reference	-0.03 (-0.05 to -0.01)	0.33 (0.31 to 0.35)
Low mobility	Reference	0.00 (-0.02 to 0.03)	0.15 (0.12 to 0.17)
RRR (multiplicative scale)			
Medium mobility	Reference	0.96 (0.94 to 0.98)	1.25 (1.23 to 1.28)
Low mobility	Reference	0.97 (0.95 to 0.99)	1.07 (1.05 to 1.10)
**Alcoholic liver disease (n = 2473)**			
High mobility	1 [Reference]	1.28 (1.25 to 1.31)	1.39 (1.37 to 1.42)
Medium mobility	1.24 (1.22 to 1.27)	1.43 (1.40 to 1.45)	1.78 (1.74 to 1.81)
Low mobility	1.44 (1.41 to 1.48)	1.78 (1.75 to 1.82)	1.86 (1.83 to 1.90)
**Interaction**			
RERI (additive scale)			
Medium mobility	Reference	-0.10 (-0.13 to -0.06)	0.14 (0.11 to 0.17)
Low mobility	Reference	0.06 (0.03 to 0.10)	0.02 (-0.01 to 0.06)
RRR (multiplicative scale)			
Medium mobility	Reference	0.90 (0.87 to 0.92)	1.02 (1.00 to 1.05)
Low mobility	Reference	0.97 (0.94 to 0.99)	0.92 (0.90 to 0.95)

Abbreviations: ARR, adjusted risk ratio (adjusting for all covariates); RERI, relative excess risk due to interaction (interaction on additive scale); RRR, ratio of risk ratio (interaction on multiplicative scale).

© 2023 Kuo CT et al. *JAMA Network Open.*

eTable 3. Interactions of Continuous Income Inequality and Social Immobility With Cause-Specific Deaths Among the Non-Hispanic White Population

Characteristic	ARR (95% CI)		
	**Suicide (n = 2750)**	**Drug overdose (n = 2438)**	**Alcoholic liver disease (n = 2473)**
Income inequality	1.08 (1.07–1.08)	1.07 (1.07–1.07)	1.13 (1.13–1.14)
Social immobility[a]	1.16 (1.16–1.17)	1.27 (1.27–1.28)	1.25 (1.24–1.25)
Product term	1.01 (1.00–1.01)	1.07 (1.06–1.07)	1.02 (1.02–1.03)
Interaction			
RERI (additive scale)	0.02 (0.02–0.03)	0.11 (0.10–0.12)	0.06 (0.06–0.07)
RRR (multiplicative scale)	1.01 (1.00–1.01)	1.07 (1.06–1.07)	1.02 (1.02–1.03)

Abbreviations: ARR, adjusted risk ratio (adjusting for all covariates); RERI, relative excess risk due to interaction (interaction on additive scale); RRR, ratio of risk ratio (interaction on multiplicative scale).

[a] Social mobility was inversely calculated as social immobility (calculated as 100 - absolute social mobility).

© 2023 Kuo CT et al. *JAMA Network Open.*
