# Supplementary material for: County-Level Income Inequality, Social Mobility, and Deaths of Despair in the US, 2000-2019
Source: JAMA Netw Open. 2023 Jul 12;6(7):e2323030. doi: 10.1001/jamanetworkopen.2023.23030 (PMC10339154; doi:10.1001/jamanetworkopen.2023.23030)
Supplement: Supplement 2. — Data Sharing Statement [file jamanetwopen-e2323030-s002.pdf]

Data Sharing Statement

Kuo. County-Level Income Inequality, Social Mobility, and Deaths of Despair in the US, 2000-2019. *JAMA Netw Open*. Published July 12, 2023. doi:10.1001/jamanetworkopen.2023.23030

Data
Data available: No

Additional Information
Explanation for why data not available: This study only used de-identified publicly available aggregate data.
